# Dynamical entropy production in cortical circuits with different network topologies

**DOI:** 10.1186/1471-2202-14-S1-P421

**Published:** 2013-07-08

**Authors:** Rainer Engelken, Michael Monteforte, Fred Wolf

**Affiliations:** 1 Max Planck Institute for Dynamics and Self-Organization, Göttingen, Germany; 2 Bernstein Center for Computational Neuroscience, Göttingen, Germany

## 

The prevailing explanation for the irregularity of spike sequences in the cerebral cortex is a dynamic balance of excitatory and inhibitory synaptic inputs - the socalled balanced state [1].

Nevertheless its statistical properties are well described by a mean field theory that is independent of the single neuron dynamics, its dynamics is far from being understood. Recently it was found that the stability of the balanced state dynamics depends strongly on the detailed underlying dynamics of individual neurons. Inhibitory networks of leaky integrate-and-fire neurons show stable chaos [2,3], while a balanced network of neurons with an active spike generation mechanism exhibits deterministic extensive chaos [4].

Previous studies of the dynamics of the balanced state used random (ErdŐs-Rényi) networks. We extended this analysis to arbitrary network topologies and analyzed the entropy production in small world topologies [5], ring networks [6], clustered networks [7], multi-layered networks [8] and topologies with different frequencies of certain network motifs [9]. We derived an analytical expression for the single spike Jacobian containing elements of the coupling matrix, which enabled us to calculate the full Lyapunov spectrum for any desired topology. Using a single neuron model in which action potential onset rapidness [10] and synaptic time constant are adjustable, we simulated the dynamics in numerically exact event-based simulations and calculated Lyapunov spectra, entropy production rate and attractor dimension for a variety of connectivities. We found that the importance of the internal single neuron dynamics for the network stability persists in different topologies. While the Entropy production and Attractor Dimension in clustered [7] and ring networks was very similar to random networks, these dynamical properties were changed substantially when introducing second order network motifs or a small world topology.

## References

[B1] van Vreeswijk C Sompolinsky H Chaos in neuronal networks with balanced excitatory and inhibitory activity Science 1996 274 1724 1726 10.1126/science.274.5293.1724 8939866

[B2] Jahnke S Memmesheimer R-M Timme M How Chaotic is the Balanced State? Frontiers in Computational Neuroscience 2009 3 13 19936316 10.3389/neuro.10.013.2009 PMC2779095

[B3] Zillmer R Brunel N Hansel D Very long transients, irregular firing, and chaotic dynamics in networks of randomly connected inhibitory integrate-and-fire neurons Physical Review E 2009 79 031909 10.1103/PhysRevE.79.031909 19391973

[B4] Monteforte M Wolf F Dynamical entropy production in spiking neuron networks in the balanced state Physical Review Letters 2010 105 268104 21231716 10.1103/PhysRevLett.105.268104

[B5] Watts Duncan J Strogatz Steven H Collective dynamics of 'small-world' networks Nature 1998 393 6684 440 442 10.1038/30918 9623998

[B6] van Vreeswijk C Sompolinsky H Chow C, Gutkin B, Hansel D, abd J Dalibard CM Irregular activity in large networks of neurons 2005 Les Houches Lectures LXXX on Methods and models in neurophysics. London: Elsevier 341 402

[B7] Ashok Litwin-Kumar Brent Doiron Slow dynamics and high variability in balanced cortical networks with clustered connections Nature Neuroscience 2012 DOI: 10.1038/nn.3220 10.1038/nn.3220 PMC4106684 23001062

[B8] Potjans TC Diesmann M 2011 arXiv:1106.5678v1 [q-bio.NC]

[B9] Zhao L Beverlin B II Netoff T Nykamp DQ Synchronization from second order network connectivity statistics Frontiers in Computational Neuroscience 2011 5 28 21779239 10.3389/fncom.2011.00028 PMC3134837

[B10] Monteforte M Wolf F Single cell dynamics determine strength of chaos in collective network dynamics Twentieth Annual Computational Neuroscience Meeting: CNS2011 2011

